# A Case of Endoscopic Partial Transverse Process and Sacral Alar Resection for Bertolotti’s Syndrome and Continued Basketball Playing Two Years After Surgery

**DOI:** 10.7759/cureus.62182

**Published:** 2024-06-11

**Authors:** Masaki Tatsumura, Tokio Kawamura, Ayako Uchida, Toru Funayama

**Affiliations:** 1 Department of Orthopaedic Surgery and Sports Medicine, Tsukuba University Hospital Mito Clinical Education and Training Center/Mito Kyodo General Hospital, Mito, JPN; 2 Department of Orthopaedic Surgery, University of Tsukuba, Tsukuba, JPN

**Keywords:** articulation of the transverse process with the sacral alar and iliac crest, minimum invasive surgery, bone resection, low back pain, bone regeneration, basketball, castellvi classification iia, intraoperative electromyography, endoscopic surgery, bertolotti’s syndrome

## Abstract

Bertolotti’s syndrome is a syndrome in which the transverse process of the most caudal lumbar vertebra becomes enlarged and articulates with the sacral alar, causing back pain. Here, we report a case of an adolescent basketball player with Bertolotti’s syndrome who was unable to resume playing despite conservative treatment and underwent an endoscopic partial transverse process and sacral alar resection. A 16-year-old male basketball player presented to our hospital with a chief complaint of left low back pain during exercise and prolonged sitting for over one month. No obvious neurological abnormality was found. X-rays and CT showed lumbosacral transitional vertebrae, and the left transverse process of the sixth lumbar vertebra articulated with the sacrum and iliac, which was the Castellvi classification IIA. A block injection into the articulated surface produced improvement in pain, but the effect was not sustained. Since the patient was refractory to conservative treatments, such as medication and physiotherapy, surgery was performed. During surgery, the articulated transverse process and sacral alar were partially resected endoscopically. Because of the proximity of the resection site to the S1 nerve root, intraoperative electromyography (free-run EMG) was used to detect nerve root irritation symptoms in real time. The patient had no postoperative complications, his low back pain improved immediately, and he returned to play basketball three months after surgery. One year after surgery, the bone resection site showed gradual bone regeneration, and two years after surgery, the transverse process and sacral alar showed a bony bridge. The transverse process was enlarged compared to immediately after surgery but remained smaller than that before surgery. The patient continued to play basketball for two years after surgery without back pain, and no symptoms due to bone regeneration appeared. In the present case, a partial resection of the transverse process and sacral alar was performed with good results. Because the bone resection site was close to the S1 nerve root, the use of an endoscope and intraoperative free-run EMG allowed for a safer procedure during the bone resection. In addition, the patient did not present with symptoms that would affect his basketball performance, although the bone regenerated and bridging occurred between the transverse process and sacral alar over a two-year postoperative course.

## Introduction

Lumbar spondylolysis is the most common cause of low back pain in adolescents [1], and it was reported that half of adolescent athletes who come to the hospital with low back pain as the chief complaint have lumbar spondylolysis [2]. Other common causes of low back pain in adolescents include sacral alar fatigue fractures [3] and Bertolotti's syndrome [4].

Bertolotti’s syndrome is a congenital lumbosacral transitional vertebral deformity associated with low back pain, first described by Bertolotti in 1917 [4]. Castellvi et al. classified the shapes of the transverse process of the lumbar spine [5], and types II and IV are considered to be causally related to low back pain [6].

Since there are no specific physical findings in adolescent low back pain, imaging inspection is very important. CT and MRI scans are mandatory for detecting the cause of low back pain in adolescents.

In the present study, an adolescent basketball player with Bertolotti’s syndrome who was unable to play despite conservative treatment underwent an endoscopic partial transverse process and sacral alar resection. We report a case in which the patient returned to play basketball after surgery and remained asymptomatic for two years.

## Case presentation

A 16-year-old male basketball player presented to our hospital with a chief complaint of low back pain that had persisted over one month. He had severe pain in the left lumbar region with exercise and prolonged sitting. 

**Figure 1:**
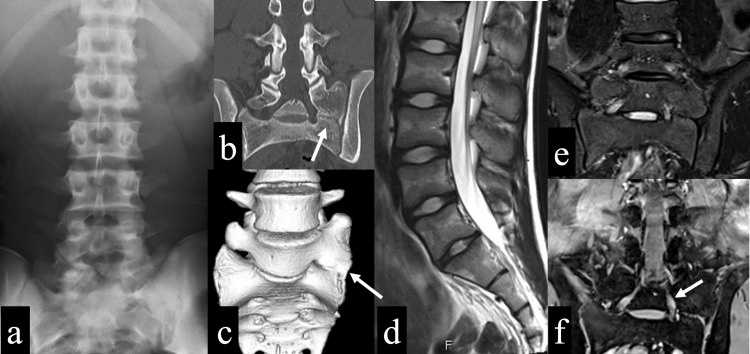
Imaging findings at the initial examination a: Plain X-ray anteroposterior view showed an enlargement of the left transverse process of the sixth lumbar vertebra. b: CT coronal view showed the left transverse process of the sixth lumbar vertebra articulating with the sacral alar and iliac bone (white arrow). c: CT 3D image showed that the left transverse process of the sixth lumbar vertebra was continuous with the sacral alar (white arrow). d: MRI T2-weighted sagittal view showed no intervertebral disc degeneration. e: MRI STIR coronal view showed no fatigue fracture of the sacral alar. f: MRI T2-weighted coronal view showed abnormal left S1 nerve root migration (white arrow).

There was no limitation of forward flexion or backward flexion with regard to the range of motion. There was no limitation in lateral flexion, but left back pain could be presented with left lateral bending. There was no sensory disturbance or motor paralysis. Deep tendon reflexes were normal. No obvious neurological abnormality was found. X-rays and CT showed a transitional vertebra, and the left transverse process of the sixth lumbar vertebra articulated with the sacral alar and the iliac crest, which was the Castellvi classification IIA (Figure 1a-1c). MRI showed no disc degeneration, lumbar spondylolysis, or fatigue fracture of the sacral alar, but it did show abnormal nerve root migration (Figure 1d-1f). 

**Figure 2:**
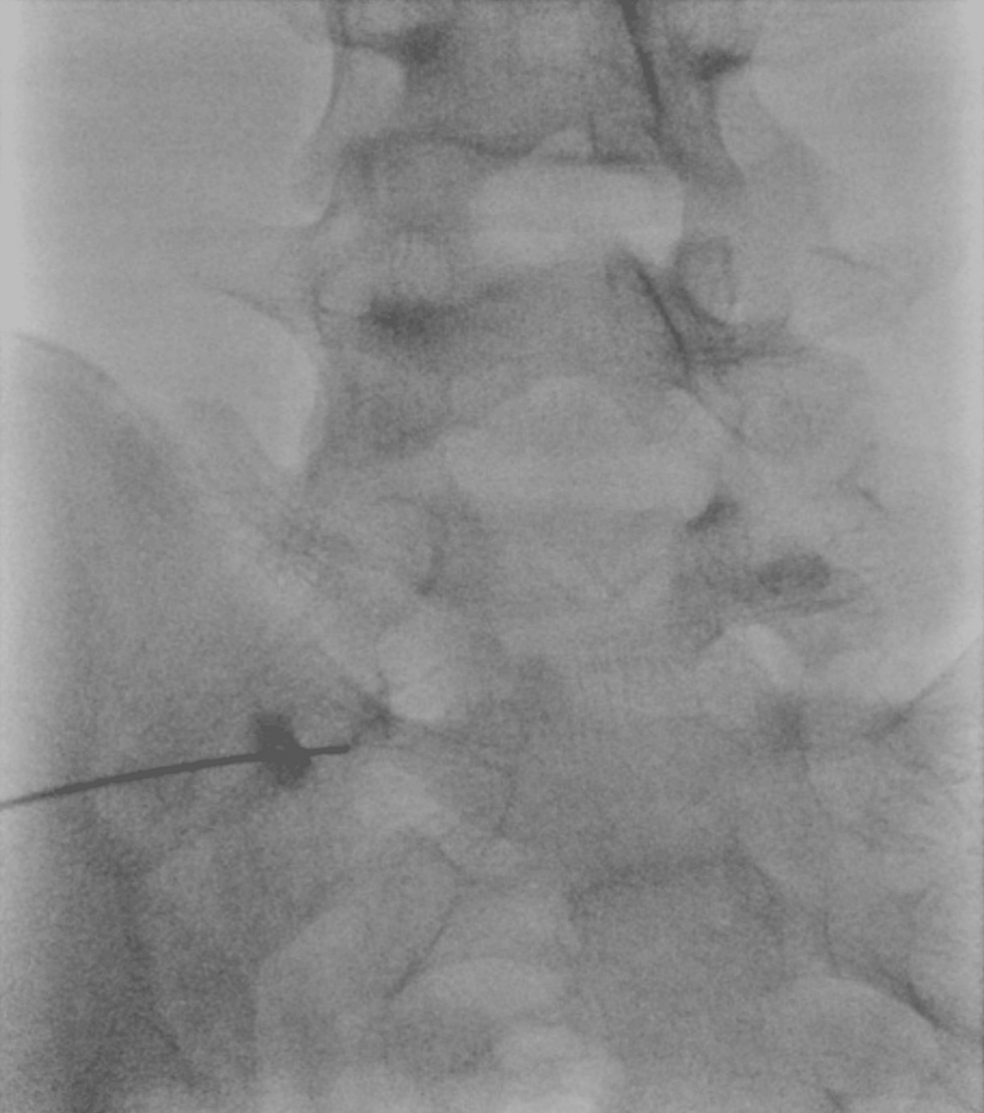
Imaging finding of block injection Contrast-containing block injection was performed on the articulated site between the transverse process and sacral alar.

The patient was refractory to conservative treatments, such as medication and physiotherapy. Block injection into the articulated site improved the pain temporally, but the effect was not sustained (Figure 2). 

**Figure 3:**
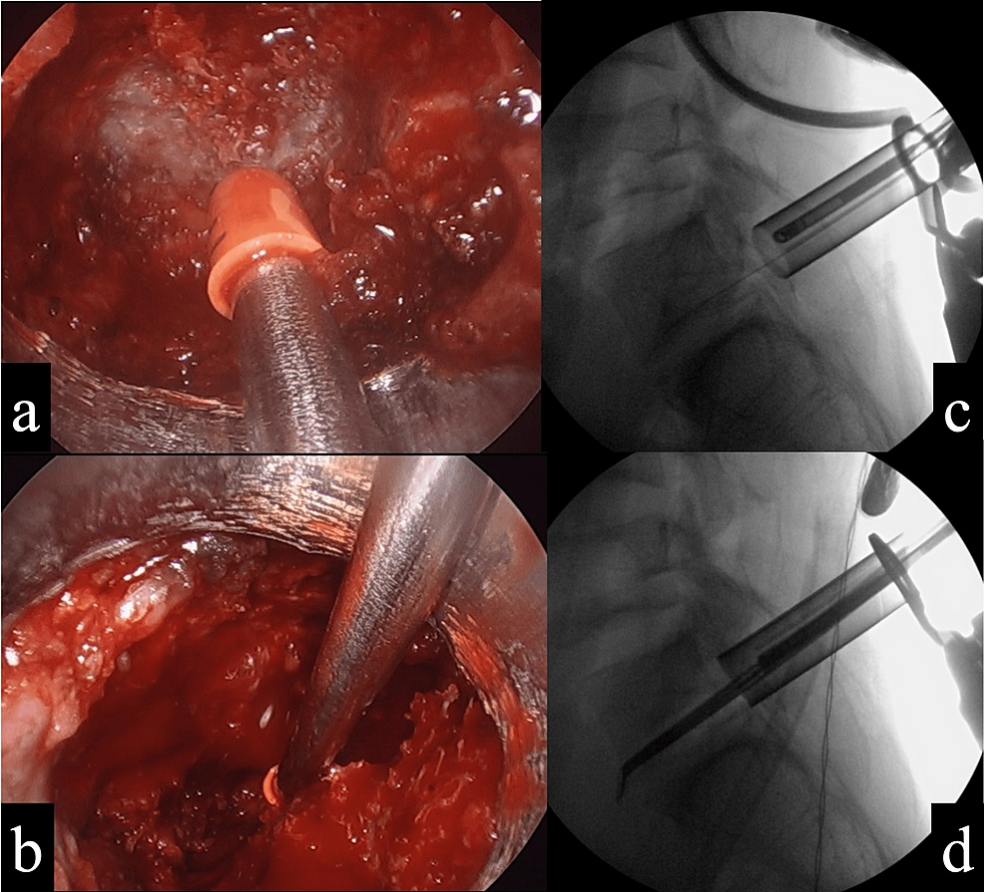
Surgical findings a: Endoscopic bone resection of the dorsal site of the transverse process. b: Bone resection was performed anterior to the sacrum under endoscopy. c: Resection was performed while confirming the endoscopic position between the transverse process and sacral alar in the fluoroscopic lateral view. d: Fluoroscopic lateral view confirmed that the resection reached the anterior sacrum.

Since the patient was refractory to conservative treatment, surgery was performed. Surgery was performed endoscopically using a tubular retractor to remove the articulated transverse process and sacral alar (Figure 3a, 3b). Because the resection extended anteriorly, fluoroscopic lateral views were also used (Figure 3c, 3d). Bone wax was used on the osteotomy surface. Because of the proximity of the resection around the S1 nerve root, intraoperative electromyography (free-run EMG) was used to detect nerve root irritation symptoms in real time while performing the surgery. 

**Figure 4:**
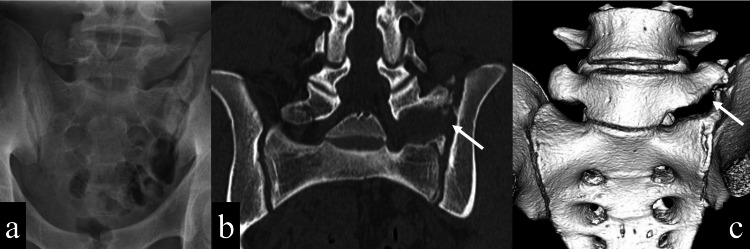
Immediate postoperative imaging findings a: Plain X-ray anteroposterior view showed dissection of the bony continuity of the transverse process and the sacral alar or iliac crest. b: CT coronal view image showed adequate bone resection (white arrow). c: CT 3D image showed dissection of the bony continuity between the transverse process and the sacral alar or iliac crest (white arrow).

The transverse process and the articular surface of the sacral alar were resected adequately without perioperative complications (Figure 4a-4c). 

On the second postoperative day, physical therapy was introduced and limb exercises were started. On the seventh postoperative day, when the patient's pain at rest disappeared and the pain was presented during movement only, trunk muscle training without movement of the lumbar spine was started. Stretching exercises with lumbar spine movements were started on the fourth postoperative week after the pain during movement disappeared. As mentioned above, the intensity was increased step by step, leading to the return to sports. The patient's back pain improved and he returned to playing basketball three months after surgery.

**Figure 5:**
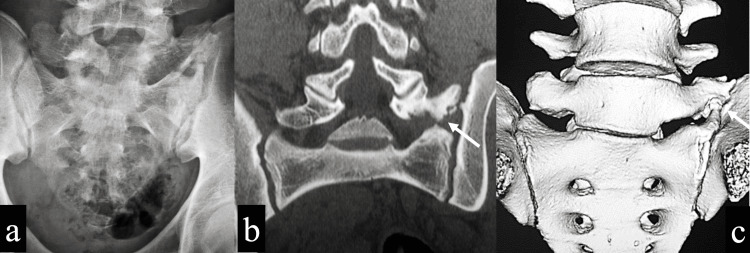
One-year postoperative imaging findings a: Plain X-ray anteroposterior view showed bony regeneration around the transverse process. b: CT coronal view showed bone regeneration of the transverse process and sacral alar (white arrow). c: CT 3D image showed no bony continuity of the transverse process and sacral alar or iliac crest (white arrow).

Thereafter, the transverse process resection area showed gradual bone formation, and plain X-rays and CT one year after surgery showed bone regeneration between the left L6 transverse process and sacral alar (Figure 5a-5c).

**Figure 6:**
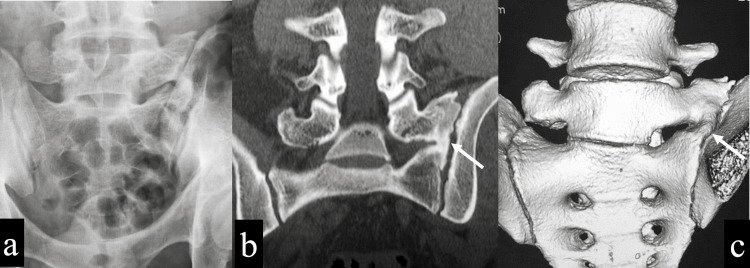
Imaging findings two years after surgery a: Plain anteroposterior X-ray showed further bony regeneration around the transverse process. b: CT coronal view showed bony continuity of the transverse process and sacral alar (white arrow). c: CT 3D image showed bony continuity of the transverse process and sacral alar (white arrow).

Finally, bone regeneration and bridging between the transverse process and sacral alar was observed, although the enlargement of the transverse process was shown compared to immediately after surgery and less pronounced than preoperatively (Figure 6a-6c). The patient never experienced back pain and continued to play basketball for two years postoperatively.

## Discussion

Treatment strategies for refractory Bertolotti’s syndrome include local injection of anesthesia and/or corticosteroids, followed by radiofrequency treatment and surgery if ineffective [7]. There are some surgical reports of decompression [8] or fusion [9] of the articulated site. Lumbosacral intervertebral fusion is as effective as decompression, but the disadvantages are loss of lumbosacral mobility and more invasiveness.

Li et al. suggested that immobilization should be considered when there is intervertebral disc degeneration between the most caudal lumbar vertebra and the sacrum [10]. Thus, decompression with transverse process excision is the first choice for younger patients without intervertebral disc degeneration to preserve the mobile lumbosacral segment. 

Endoscopic decompression is a minimally invasive and safe procedure [11]. However, there is a risk of nerve root injury associated with the surgical procedure because of the proximity of nerve roots, as in far-out syndrome. This presented case has the migrated S1 nerve root, and we had to strategize to avoid nerve injury. In this case, the use of an endoscope during transverse process resection allowed for safe surgery with a magnified view of the surgical site. In addition, the EMG free-run was used during the surgery to detect nerve root irritation symptoms in real time. Moreover, we could perform partial resection of the transverse process and sacral alar with endoscopy to ensure adequate resection.

Short-term results after endoscopic surgery are scattered [7,12]. There are no reports describing bone regeneration in the resected site in short-term case reports. In our case, bone regeneration of the resected transverse process and sacral alar was observed after two years postoperatively, but it was not symptomatic enough to affect athletic performance. The bony bridge between the transverse process and the sacrum has stabilized and is the cause of the asymptomatic condition during sports.

Differential diagnosis is important because fatigue fractures of the sacral alar can occur in adolescent athletes [3].

## Conclusions

An adolescent basketball player with Bertolotti’s syndrome who was unable to play despite conservative treatment underwent endoscopic partial resection of the transverse process and sacral alar. In this case, the bone resection was performed endoscopically with intraoperative electromyography, and pain relief was obtained immediately after surgery. After more than two years of postoperative follow-up, the bone regenerated and bridging occurred between the transverse process and sacral alar, but the patient had never felt back pain and did not present any problems in playing basketball.

## References

[1] Micheli LJ, Wood R (1995). Back pain in young athletes. Significant differences from adults in causes and patterns. Arch Pediatr Adolesc Med.

[2] Tatsumura M, Gamada H, Ishimoto R (2018). Prevalence of curable and pseudoarthrosis stages of adolescent lumbar spondylolysis. J Rural Med.

[3] Tatsumura M, Eto F, Nagashima K (2021). Features of sacral alar fatigue fractures in adolescent athletes with overuse. Sci Rep.

[4] Holm EK, Bünger C, Foldager CB (2017). Symptomatic lumbosacral transitional vertebra: a review of the current literature and clinical outcomes following steroid injection or surgical intervention. SICOT J.

[5] Castellvi AE, Goldstein LA, Chan DP (1984). Lumbosacral transitional vertebrae and their relationship with lumbar extradural defects. Spine (Phila Pa 1976).

[6] Nardo L, Alizai H, Virayavanich W (2012). Lumbosacral transitional vertebrae: association with low back pain. Radiology.

[7] Chitneni A, Kim R, Danssaert Z, Kumar S (2024). A proposed treatment algorithm for low back pain secondary to Bertolotti's syndrome. Pain Physician.

[8] Ugokwe KT, Chen TL, Klineberg E, Steinmetz MP (2008). Minimally invasive surgical treatment of Bertolotti's syndrome: case report. Neurosurgery.

[9] Santavirta S, Tallroth K, Ylinen P, Suoranta H (1993). Surgical treatment of Bertolotti's syndrome. Follow-up of 16 patients. Arch Orthop Trauma Surg.

[10] Li Y, Lubelski D, Abdullah KG, Mroz TE, Steinmetz MP (2014). Minimally invasive tubular resection of the anomalous transverse process in patients with Bertolotti's syndrome: presented at the 2013 Joint Spine Section Meeting: clinical article. J Neurosurg Spine.

[11] Shibayama M, Ito F, Miura Y, Nakamura S, Ikeda S, Fujiwara K (2011). Unsuspected reason for sciatica in Bertolotti's syndrome. J Bone Joint Surg Br.

[12] Stein E, Panjeton GD, Kumar S (2023). Endoscopic resection of pseudoarticulation as a treatment for Bertolotti's syndrome. Cureus.

